# Dilated ascending aorta: is reductive ascending aortoplasty really forgotten?

**DOI:** 10.1186/1749-8090-8-S1-P6

**Published:** 2013-09-11

**Authors:** Z Jonjev, M Rosic, B Mihajlovic, A Redzek, M Fabri

**Affiliations:** 1 Institute for CVD of Vojvodina, Sremska Kamenica, Serbia

## Background

Patients with dilated ascending aorta require surgical correction due to increased risk for aortic dissection and/or rupture. However, there is a controversy whether the dilated ascending aorta should be replaced with prosthetic graft or reduced by reductive ascending aortoplasty (RAA). The aim of this study is to analyze our results with “cut and sew” unwrapped RAA.

## Method

There were 25 pts operated on from 2002 to 2013. The average size of aorta was 5.5±0.4cm, and most of the patient had concomitant aortic valve stenosis or insufficiency. All patients were treated with unwrapped RAA.

## Results

There was no postoperative (30 days) mortality. The average size of reduced aorta was 3.4±0.3cm. There was no significant postoperative bleeding or other major immediate morbidities. Most of the patients had abnormal histological finding of the aortic wall specimen. Recurrence rate after 10 years follow up was 4.5% (1pt).

## Conclusion

Unwrapped RAA procedure could be successfully applied in selective, preferable older patients with dilation of ascending aorta. However, prosthetic graft implantation is recommended in patients where rapid recurrent aortic dilation is expected.

